# The Role of Ketamine as a Component of Multimodal Analgesia in Burns: A Retrospective Observational Study

**DOI:** 10.3390/jcm13030764

**Published:** 2024-01-29

**Authors:** Marina Stojanović, Milana Marinković, Biljana Miličić, Milan Stojičić, Marko Jović, Milan Jovanović, Jelena Isaković Subotić, Milana Jurišić, Miodrag Karamarković, Aleksandra Đekić, Kristina Radenović, Jovan Mihaljević, Ivan Radosavljević, Branko Suđecki, Milan Savić, Marko Kostić, Željko Garabinović, Jelena Jeremić

**Affiliations:** 1 Faculty of Medicine, University of Belgrade, 11000 Belgrade, Serbia; 2 Center for Anesthesiology and Resuscitation, University Clinical Center of Serbia, 11000 Belgrade, Serbia; 3 Clinic for Burns, Plastic and Reconstructive Surgery, University Clinical Center of Serbia, 11000 Belgrade, Serbia; 4 Department of Medical Statistics and Informatics, School of Dental Medicine, University of Belgrade, 11000 Belgrade, Serbia; 5 Clinic for Thoracic Surgery, University Clinical Center of Serbia, 11000 Belgrade, Serbia

**Keywords:** acute pain control, burns, burn wound dressing, multimodal analgesia, opioid consumption, postoperative analgesia, postoperative pain, ketamine

## Abstract

**Background**: Burn wound dressing and debridement are excruciatingly painful procedures that call for appropriate analgesia—typically multimodal. Better post-procedural pain management, less opioid use, and consequently fewer side effects, which could prolong recovery and increase morbidity, are all benefits of this type of analgesia. Intravenously administered ketamine can be effective as monotherapy or in combination with opioids, especially with procedural sedation such as in burn wound dressing. **Methods**: This observational study investigated the effect of ketamine administered in subanesthetic doses combined with opioids during burn wound dressing. The study was conducted from October 2018 to October 2021. A total of 165 patients met the inclusion criteria. A total of 82 patients were in the ketamine group, while 83 patients were dressed without ketamine. The main outcome was the effect of ketamine on intraprocedural opioid consumption. The secondary outcome included the effect of ketamine on postprocedural pain control. **Results**: Patients dressed with ketamine were significantly older (*p* = 0.001), while the mean doses of intraoperatively administered propofol and fentanyl were significantly lower than in patients dressed without ketamine (150 vs. 220 mg, *p* < 0.001; and 0.075 vs. 0.150 mg, *p* < 0.001; respectively). **Conclusions**: Ketamine was an independent predictor of lower intraoperative fentanyl consumption, according to the multivariate regression analysis (*p* = 0.015). Contrarily, both groups of patients required postoperative tramadol treatment, while intraoperative ketamine administration had no beneficial effects on postoperative pain management.

## 1. Introduction

Burns present some of the most painful injuries; thus, pain control in such trauma remains challenging and demanding [1]. The importance of effective acute burn pain management is reflected in the reduction in acute suffering as well as the prevention of neuropathic and chronic pain [2].

When selecting an analgesic, it is important to consider the changes that take place during the acute phase of a burn injury. These changes include decreased blood flow through tissues, which lowers drug clearance, changes in acute phase protein concentration, which affects drug binding to proteins, as well as metabolic changes and volume shifts [1]. The American Burn Association Guidelines (ABA) state that a variety of drugs, including opioids, non-steroidal anti-inflammatory drugs (NSAIDs), gabapentin, pregabalin, alpha-2 agonists, lidocaine, and ketamine, can be used to treat pain in burn patients [1]. Lidocaine given intravenously ought to be used as a second- or third-line adjuvant analgesic [1].

Burn wound dressing and debridement are extremely painful procedures that call for appropriate analgesia—typically multimodal. The combination of medications with various but complementary or additive mechanisms of action is suggested by the multimodal analgesia principle in order to provide the optimal pain management [3]. Better post-procedural pain management, less opioid use, and consequently fewer side effects, which could prolong recovery and increase morbidity, are all benefits of this type of analgesia [3,4,5]. Despite studies that demonstrate the effectiveness of multimodal analgesia in reducing postoperative pain, it is important to consider both the patient’s specific pain response and the pain mechanism particular to the treatment when selecting non-opioid medications [5].

There exists a growing interest in the use of ketamine for the management of acute pain to reduce the dose-dependent adverse effects of opioids [1]. Ketamine induces dissociative anesthesia with effects of sedation, amnesia, and pain relief [6]. Intravenously administered ketamine can be effective as monotherapy or in combination with opioids, especially with procedural sedation such as in burn wound dressing, as it is a non-competitive N-methyl d-aspartate (NMDA) receptor antagonist with anesthetic, analgesic, anti-inflammatory, and antidepressant effects [7,8,9]. Ketamine is a drug of choice for short-term procedures when muscle relaxation is not required [10]. A patient’s management of acute trauma pain might be complicated due to the patient’s mental state, age, or alteration in their state of consciousness [11]. Ketamine is frequently used in severely injured patients and appears to be safe in this group. It has been widely used for emergency surgery in field conditions in war zones [12]. A 2011 clinical practice guideline supports the use of ketamine as a sedative in emergency medicine, including during physically painful procedures [13].

Another benefit of ketamine is reflected in its characteristic of not inducing hypotension or bradycardia [14,15]. Ketamine releases catecholamines by inhibiting neuronal and extraneuronal reuptake, raising cardiac output, heart rate, and arterial pressure [15]. White et al. found that during anesthesia induction ketamine increased mean arterial pressure by 10% [15]. According to clinical practice guidelines, ketamine is the drug of choice for people in traumatic shock who are at risk of hypotension [13]. It is also frequently used to provide analgesia and anesthesia to patients with hemodynamic instability and to morbidly obese patients who require high opioid doses [3,16]. Burn shock incorporates distributive, hypovolemic, and cardiogenic features, and is characterized by a diffuse capillary leak in which electrolytes, proteins, and plasma decrease the volume of the circulatory system, as well as interfere with end-organ perfusion, leading to cellular hypoxia [17,18,19]. Considering the circulatory changes as well as the high risk of hemodynamic instability, the use of ketamine for short-term procedures such as wound dressing in burn patients would have great benefits. Even though it has been used as an anesthetic for years, there is a lack of data showing the effect of subanesthetic doses of ketamine combined with opioids during burn wound dressing. Thus, further studies are still needed to elucidate their indications in this context.

The aims of this study are to examine the effect of ketamine administered during burn wound dressing on intraprocedural opioid consumption, particularly its effect in relation to burn size and depth, as well as its effect on postprocedural pain control.

## 2. Materials and Methods

### 2.1. Study Design and Population

**Figure 1:**
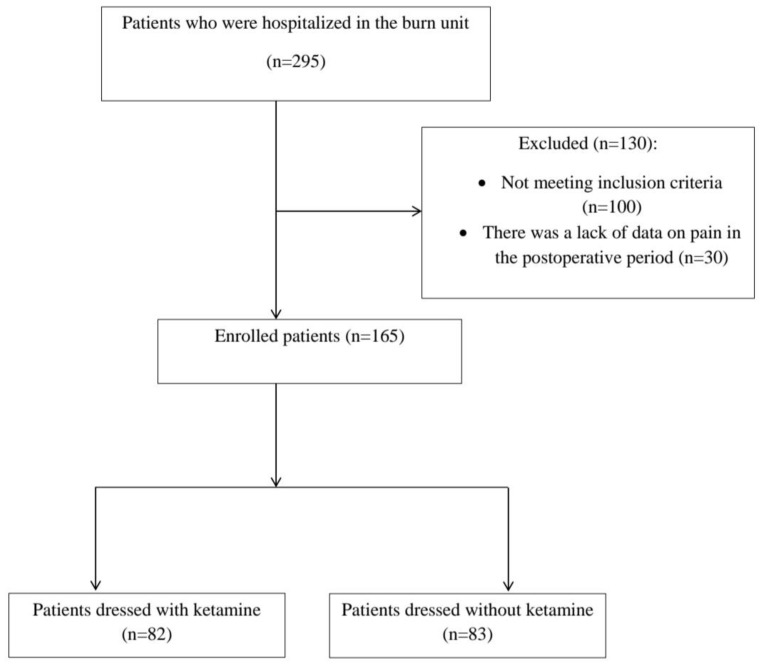
Flow chart of the study. A total of 130 of the 295 patients were excluded from the study. A total of 165 of patients met the inclusion criteria. A total of 82 of the included patients were dressed with ketamine, while 83 were dressed without ketamine.

This study was designed as a retrospective observational study and included patients with burn injuries treated at the Clinic for Burns, Plastic, and Reconstructive Surgery, University Clinical Centre of Serbia in Belgrade in the period from October 2018 to October 2021. The study was approved by the Institutional Review Board (date 20 September 2021, approval number 415/66) and performed in accordance with the tenets of the Declaration of Helsinki. Inclusion criteria were patients of both sexes, older than 15 years, with burn injuries of various extents, who required burn wound dressings and debridement in the operating room under intravenous anesthesia between the 3rd and the 5th day post-injury (following the initial 48 h of hemodynamic stabilization and before the planned definitive surgical treatment). Patients with superficial partial-thickness burns, deep partial-thickness burns, as well as patients with a combination of deep and superficial burns, were included. Of the 295 patients admitted to the burn unit, 165 met the inclusion criteria for the study while 130 patients were excluded from the study. Patients with inhalation injuries, intubated patients on mechanical ventilation, and patients with well-known contraindications for the use of ketamine were excluded. Due to the pathophysiological response to pain in deep burns, patients with full-thickness burns were also excluded from the study. Another exclusion criterion was insufficient postoperative pain data (Figure 1).

### 2.2. Anesthetic Management

The choice of anesthetic management was decided by the senior anesthesiologists. All patients received premedication of 0.1 mg/kg midazolam and 0.5 mg atropine intramuscularly, 30 min before the burn wound dressing was performed. The position of the patient on the operating table depended on the location of the burns and included a back position, a side position, and a prone position. To secure the airway, laryngeal masks of appropriate size were used, while anesthesia was maintained with a gas mixture of air (2 L/min) and oxygen (2 L/min).

Patients were divided into two groups based on whether they received ketamine during burn dressing: 82 patients in the ketamine group and 83 patients in the non-ketamine group. A subanesthetic dose of ketamine (0.5 mg/kg) was prescribed to patients in the ketamine group at the beginning of the intervention. Patients from both groups received fentanyl and propofol in a dose individually determined by their requirements during the burn wound dressing. The depth of the burn injury, the total body surface area burned (TBSA), the age and sex of the patient, the duration of the treatment, as well as the impact of the administration of subanesthetic doses of ketamine (0.5 mg/kg) on the intraoperative requirements for opioid analgesics (fentanyl), were noted.

After burn wound dressing was performed, the patients were admitted to the intensive care unit (ICU). Standard hemodynamic parameters (pulse rate, blood pressure, oxygen saturation), and electrocardiogram (ECG) were continuously monitored and recorded every hour during the first 24 h after the procedure. Standard laboratory variables such as blood counts, coagulation status, and biochemical analyses were checked.

Pain intensity at rest was measured using the numerical pain intensity scale (NPIS) 1 h, 3 h, 6 h, 12 h, and 24 h after wound dressing. The intensity of pain in motion was not measured, considering the fact that different parts of the body were affected by burns, and it was thus not possible to define a specific motion that would be valid for all patients. A combination of NSAIDs and paracetamol was used for postprocedural analgesia; thus, all patients received paracetamol 1 g and ketorolac 30 mg every 8 h during the first 24 h. In patients whose non-opioid analgesic therapy was not satisfactory (when the intensity of pain on the NPIS scale was over 4), tramadol was prescribed in a dose of 100 mg, which was repeated according to the patient’s requirements. Additionally, the impact of intraprocedurally administered ketamine on the need for additional postoperative analgesia and the need for postoperative tramadol was examined.

### 2.3. Study Outcomes and Data Collection

The main outcome was assessing the effect of ketamine on intraprocedural opioid consumption, while the secondary outcome included the effect of ketamine on postprocedural pain control. Demographics, medical histories, and perioperative variable data, including the type and amount of anaesthetic used, pain intensity, as well as the need for additional analgetic, were collected from medical records. Due to the lack of information, the side effects of ketamine could not be analyzed.

### 2.4. Statistical Analysis

SPSS software for Windows version 20.0 was used for statistical data analysis. Numerical variables are shown in the form of mean values ± standard deviation, coefficient of variation, and minimum and maximum values, while categorical variables are shown as absolute numbers and percentages. Using the Kolmogorov–Smirnov test, the normality of the data distribution was checked. Pearson’s χ^2^ test (contingency tables) was used to analyze data with normal distribution, while the Mann–Whitney U test was used to analyze data without normal distribution. Predictors of differences between the analyzed groups of patients (the group without ketamine and the group with ketamine) were determined by logistic regression analysis. Values of *p* < 0.05 were considered statistically significant.

## 3. Results

Of the 165 patients included in the study, 63% were male while 37% were female. The mean age was 51.86 ± 11.42 years (the youngest patient was 15 years old, while the oldest was 93). The mean TBSA was 23.97 ± 15.45%, with the least TBSA burned being 3%, and the largest TBSA burned being 66%. According to TBSA burned, patients were divided into 6 groups (TBSA less than 10%, TBSA 11–20%, TBSA 21–30%, TBSA 31–40%, TBSA 41–50%, and TBSA above 50%). Most patients (33.9%) were in the 11–20% TBSA burned group, while the fewest (6.1%) were in the 41–50% TBSA burned group. There were about 10.3% of patients with burns greater than 50% TBSA. The patients’ characteristics are shown in Table 1.

There was no statistical significance in the difference between the genders in the ketamine and the non-ketamine group. Patients in the ketamine group were significantly younger (46.03 ± 19.59 vs. 55.38 ± 17.95, *p* = 0.001, Mann–Whitney U test). However, there were no differences between groups according to TBSA burned (*p* = 0.733, Mann–Whitney U test).

The mean body weight in the ketamine group was 71.94 ± 8.04, while in the non-ketamine group it was 72.95 ± 7.55, with no statistical difference (*p* = 0.393, Mann–Whitney U test). The median dose of intraoperatively administered propofol in the ketamine group was 150 mg (minimum 0 mg, maximum 480 mg), compared to the 220 mg (minimum 70 mg, maximum 500 mg) in the non-ketamine group, with a statistically significant difference (*p* < 0.01, Mann–Whitney U test). Additionally, patients in the ketamine group received significantly lower doses of fentanyl during wound dressing than patients in the non-ketamine group (0.075 mg vs. 0.150 mg, *p* < 0.01, Mann–Whitney U test) (Table 2).

Although rest pain intensity measured by NPIS was lower in the first 12 h after wound dressing in the ketamine group, statistical significance was not observed compared to the non-ketamine group (Table 3). About 32.7% of patients received tramadol in the postoperative period, while 67.3% of patients did not require additional analgesia. The requirement for tramadol was quite uniform in patients who received intraoperative ketamine, compared with the patients who did not receive intraoperative ketamine (*p* = 0.631, Pearson χ^2^ test) (Table 2).

The logistic regression analysis showed that patients who received ketamine during burn wound dressing were significantly older; moreover, they received significantly lower doses of both propofol and fentanyl than patients who did not receive ketamine (Table 4). Additionally, using multivariate logistic regression analysis, intraoperative use of ketamine was found to be an independent predictor of reduced intraoperative use of fentanyl (Table 5).

## 4. Discussion

This study demonstrated that when ketamine was used in subanesthetic doses (0.25–0.5 mg/kg) during burn wound dressing under intravenous anesthesia in patients with burns of various extents between the third and the fifth day after injury, the patients received lower doses of propofol and fentanyl. The study also demonstrated that ketamine was an independent predictor of decreased intraoperative fentanyl use.

In a meta-analysis, McGiness et al. demonstrated that ketamine had the best analgesic impact on burn patients when compared to other analgesics [8]. They claimed that ketamine given intravenously at a dose of 0.3 mg/kg/h considerably decreased the occurrence of secondary hyperalgesia when compared to a dose of 0.15 mg/kg/h, whereas the addition of morphine had no effect [8]. Moreover, they demonstrated that adverse effects like nausea and vomiting occurred following the injection of morphine, but sleepiness was, as anticipated, dose-dependent [8]. They did not investigate if hallucinations occurred. In this study, it was demonstrated that ketamine’s coanalgesic action might be used to effectively manage pain during burn wound dressing at subanesthetic doses. However, the occurrence of side effects from ketamine were not investigated.

In patients who underwent skin grafting, Lennertz et al. investigated how perioperative multimodal analgesia affected both intraoperative and postoperative opioid consumption [20]. They demonstrated that the most significant predictors of postoperative opioid usage were age and TBSA burned [20]. Younger patients in their study required larger morphine doses; morphine intake fell by 2.7 morphine equivalents (ME) for every year of age (1 ME = 1 mg of oral morphine) [20]. They also demonstrated that for every 10% rise in TBSA burned, there was a 1.8 ME increase in opioid intake, with the average amount of opioid use rising from 166 ± 94 ME preoperatively to roughly 218 ± 117 ME in the first 24 h postoperatively [20]. Similar to our findings, this study demonstrated that the single administration of ketamine, whether as preemptive analgesia or intraoperatively, had no impact on the postoperative reduction in opioid consumption. Patients with a larger %TBSA burned are likely to require longer time in the OR necessary for burn wound dressing, resulting in a higher consumption of fentanyl which decreases the level of pain in the early post-procedural course. In this study, however, the patients who received ketamine during wound dressing had significantly lower fentanyl use; thus, a higher level of pain in the post-procedural course would be expected. Given how this study found no difference in pain intensity after dressing between the groups, it can be assumed that ketamine had some effect on pain intensity. The injection of ketamine in a bolus dose without further continuous infusion administration may be one explanation for this. The average percent of body surface area burned was higher (8.1% vs. 23.7% TBSA), and a positive correlation was found between the %TBSA burned and the intraoperative use of opioids. This was to be expected given that patients with extensive burns required more intraoperative analgesic administration due to the longer duration of burn wound dressing and the intensity of their pain.

Ketamine was utilized by Brennan et al. in a bolus dose of 1.2 mg/kg, repeated as needed for 5 min during burn wound dressing, along with benzodiazepines at an average dose of 3 mg, and opioids (fentanyl at an average dose of 10 ME) in 26% of patients [21]. Dysphoric reactions were seen in 6% of individuals, whereas the remaining 6% experienced ketamine-induced hypertension that responded positively to labetalol given intravenously [11]. They used a higher dose of ketamine (0.25–0.5 mg/kg vs. 1.2 mg/kg) and did not utilize propofol, which increased the risk of complications such as ketamine-induced hypertension [21]. In order to achieve the desired level of analgosedation during wound dressing in burn patients, Gündüz et al. investigated the effects of various drug combinations (1 mg/kg ketamine followed by 1 mg/kg dexmedetomidine, 0.05 mg/kg midazolam, and saline solution) [22]. The dexmedetomidine–ketamine combination performed better than other combinations in terms of blood pressure and heart rate, as well as analgesia and postoperative sedation duration [22]. In addition, Zor et al. analyzed the ideal analgesic combination for the dressing of burn wounds [23]. The participants in their study were split into three groups: the first received a dose of 2 mg/kg of ketamine alone; the second, 1 mg/kg of tramadol and, after 30 min, 1 g/kg of dexmedetomidine and 2 mg/kg of ketamine; and the third, 1 mg/kg of tramadol and, after 30 min, 0.05 mg/kg of midazolam and 2 mg/kg of ketamine [23]. With regard to pain management and side effects, the second group performed better [23].

Ketamine can be taken orally, rectally, or intranasally in addition to intravenously. There have been some investigations into ketamine taken orally [24,25]. Kundra et al. investigated the effects of oral ketamine (5 mg/kg) and dexmedetomidine (4 g/kg) on pain management during burn wound dressing [24]. They showed noticeably lower pain scores, with 67% in the ketamine group and 44% in the dexmedetomidine group (the mean for the groups being 2.6 ± 0.6 and 3.8 ± 0.8, respectively) [24]. In a retrospective study, Lintner et al. found that patients who received oral ketamine in doses of 0.5–3 mg/kg with 2–4 mg of midazolam as premedication had statistically significantly lower doses of intravenously administered opioids (fentanyl or hydromorphine, 50 mg vs. 75 mg, *p* = 0.009) [25]. Ketamine, whether administered orally or intravenously, reduces the requirement for intraoperative opioid consumption and, as a result, the likelihood of side effects associated with the use of opioids in large doses. Furthermore, according to Grossmann et al., children with burns less than 6% TBSA burned can achieve a satisfactory level of analgosedation with a reasonable recovery period and few side effects when receiving rectally administered ketamine at a dose of 6 mg/kg combined with 0.5 mg/kg of dormicum [26].

The effectiveness of ketamine as a coanalgesic in postoperative pain control following various types of surgery, as well as the prevalence of side effects, were evaluated by Subramaniam et al. in a meta-analysis and systematic review [27]. A total of 20 out of the 37 studies included in the analysis revealed that the addition of ketamine to opioids had a positive impact on postoperative pain management, particularly when it was given as a bolus or continuous infusion [27]. It was not demonstrated, however, how the timing of ketamine administration affected its analgesic impact (before incision, during operation, or after surgery). In individuals who had developed an acute tolerance to opioids, the favorable effects of ketamine on the lowering of pain intensity and the opioid sparing effect are described in the literature [28]. The use of ketamine was considered when the severity of postoperative pain necessitated high doses of opioids, such as in major abdominal and thoracic surgery, given how a combination of local anesthetics, NSAIDs, and opioids typically provided adequate analgesia in surgical procedures like appendectomy, tonsillectomy, laparoscopic surgery, and knee arthroscopy [27]. Additionally, the administration of ketamine had no impact on the incidence of psychomimetic side effects or the reduction in respiratory depression, pruritus, or postoperative nausea and vomiting [27].

According to Brinck et al., preoperative ketamine injection considerably decreased the need for postoperative opioids by 8 ME in the first 24 h and by 13 ME in the next 48 h [29]. Additionally, pain intensity at rest decreased by 19% during the first 24 h and by 22% during the next 48 h, whereas pain intensity during movement decreased by 14% during the initial 24 h and by 16% during the subsequent 48 h [29]. In addition, administering ketamine added a 54 min delay before the administration of the first analgesic in the postoperative phase [29]. Similar outcomes were obtained by Laskowski et al., and Wang et al., but none of them demonstrated a connection between the ketamine dose delivered and the desired outcome [30,31].

This study is limited by its retrospective nature, the single-center design, as well as a small number of participants. Additionally, the study’s limitations stem from the fact that just one parameter of postprocedural rest pain was examined, which fails to take into account the multidimensional characteristics of pain pathways as well as patient-specific perceptions of pain. The use of subanesthetic doses of ketamine as part of multimodal analgesia in other, less painful procedures could produce different results in terms of even better pain control and lower postoperative pain score values, since burns are considered an extremely painful trauma and burn wound dressing and debridement are a typically very painful procedure. Furthermore, the post-procedural side effects of ketamine were not investigated in this study.

## 5. Conclusions

Administering ketamine intraoperatively in subanesthetic (analgesic) doses showed a considerably decreased need for intraoperative opioids. Patients with burns less than 20% TBSA burned as well as patients with burns larger than 21% TBSA burned both showed positive effects in terms of the reduction in use of opioids (fentanyl) and propofol. Ketamine was an independent predictor of lower intraoperative fentanyl use, according to the multivariate regression analysis. Contrarily, both groups of patients required postoperative tramadol treatment, while intraoperative ketamine administration had no statistically significant effects on postoperative pain management.

## Figures and Tables

**Table 1:** Patients’ characteristics.

Variable	N (%)
Included patients	165 (100%)
Age (mean ± SD), years	51.86 ± 11.42
Sex: male	104 (63.0%)
TBSA burned (mean ± SD)%	23.97 ± 15.45%
Patients with TBSA burned <10%	25 (15.2%)
Patients with TBSA burned 11–20%	56 (33.9%)
Patients with TBSA burned 21–30%	39 (23.6%)
Patients with TBSA burned 31–40%	18 (10.9%)
Patients with TBSA burned 41–50%	10 (6.1%)
Patients with TBSA burned >51%	17 (10.3%)

SD—standard deviation; N—number of patients; TBSA—total body surface area.

**Table 2:** Characteristics of patients dressed with and without ketamine.

Variable	With Ketamine N (%)	Without Ketamine N(%)	*p* Value
Patients	82 (49.7%)	83 (50.3%)	
Age (mean ± SD), years	46.0.3 ± 19.59	55.38 ± 17.95	0.001
Sex:			0.599
male	52 (31.5%)	52 (31.5%)	
TBSA burned (mean ± SD)%	24.75 ± 16.27	23.37 ± 14.84	0.733
TBSA% subgroups:			0.918
TBSA burned <10%	13 (7.9%)	11 (6.7%)	
TBSA burned 11–20%	26 (15.8%)	30 (18.2%)	
TBSA burned 21–30%	18 (10.9%)	21 (12.7%)	
TBSA burned 31–40%	9 (5.5%)	10 (6.1%)	
TBSA burned 41–50%	7 (4.2%)	3 (1.8%)	
TBSA burned >51%	9 (5.4%)	8 (4.8%)	
Body wight (mean ± SD), kg	71.94 ± 8.04	72.95 ± 7.55	0.393
Fentanyl median (range) mg	0.075 (0.00–0.40)	0.150 (0.05–0.50)	<0.001
Propofol, median (range) mg	150 (0–480)	220 (70–500)	<0.001
Tramadol postoperatively			0.631
Yes	30 (18.2%)	24 (14.5%)	

SD—standard deviation; N—number of patients; TBSA—total body surface area.

**Table 3:** Rest pain intensity measured with numerical pain intensity scale 1 h, 3 h, 6 h, 12 h, and 24 h after procedure. No statistical significance was observed between two groups.

Variable	With Ketamine	Without Ketamine	*p* Value
Rest pain 1 h after procedure	1.8 ± 0.2	2.2 ± 0.2	0.691
Rest pain 3 h after procedure	3.3 ± 0.7	3.6 ± 0.2	0.851
Rest pain 6 h after procedure	5.0 ± 0.2	5.4 ± 0.6	0.776
Rest pain 12 h after procedure	5.3 ± 0.1	5.5 ± 0.3	0.966
Rest pain 24 h after procedure	3.8 ± 0.4	3.7 ± 0.3	0.899

**Table 4:** Univariate regression analysis showed that patients who received ketamine during burn wound dressing were significantly older; moreover, they received significantly lower doses of both propofol and fentanyl than patients who did not receive ketamine.

Characteristics	OR (95% CI)	*p* Value
Gender	1.187 (0.626–2.252)	0.599
Age	1.027 (1.009–1.044)	0.002
TBSA burned	0.994 (0.975–1.014)	0.567
TBSA burned in subgruops	0.952 (0.775–1.170)	0.641
Body weight (kg)	1.017 (0.977–1.058)	0.410
Propofol (mg)	1.007 (1.003–1.010)	<0.001
Fentanyl	5247.123 (73.202–376,112.12)	<0.001

CI—confidence interval; TBSA—total body surface area.

**Table 5:** Multivariate regression analysis showed that intraoperative use of ketamine was an independent predictor of reduced intraoperative use of fentanyl.

Characteristics	OR (95% CI)	*p* Value
Propofol (mg)	1.005 (1.000–1.009)	0.095
Fentanyl	822.330 (3.693–183,088.334)	0.015

CI—confidence interval.

## Data Availability

The data presented in this study are available on request from the corresponding author. The data are not publicly available due to Institutional research permit.
